# The role of eosinophils and basophils in peripheral blood in liver injury: findings from a cross-sectional study of an external hospital-based dataset and the NHANES (2007–2012)

**DOI:** 10.3389/fphys.2026.1900697

**Published:** 2026-07-30

**Authors:** Shan Gao, Kewei Du, Meilong Zhang, Yue Ma, Juanhong He, Tuanjie Che, Linjing Li, Shangdi Zhang

**Affiliations:** 1 Laboratory Medicine Center, The Second Hospital & Clinical Medical School, Lanzhou University, Lanzhou, China; 2 Cuiying Biomedical Research Center, The Second Hospital & Clinical Medical School, Lanzhou University, Lanzhou, China; 3 School of Public Health, Gansu University of Chinese Medicine, Lanzhou, China; 4 Innovation Center of Functional Genomics and Molecular Diagnostics Technology of Gansu Province, Lanzhou, China

**Keywords:** basophil count, eosinophil count, liver injury, NHANES, prognostic biomarker

## Abstract

**Background:**

As a major global health concern, liver injury requires effective preventive and therapeutic measures. This study aimed to examine the relationship between eosinophil and basophil counts and liver injury.

**Methods:**

We analyzed two cohorts: 16, 328 subjects from a hospital-based dataset and 14, 962 individuals from the NHANES (2007-2012) database. After excluding cases with missing values, participants were categorized into groups with or without liver injury. Statistical analyses included weighted logistic regression (across three models), the XGBoost algorithm to identify critical factors, and restricted cubic spline analysis to explore non-linear relationships.

**Results:**

Statistical analyses revealed significant differences in eosinophil and basophil counts in peripheral blood between the liver injury and non-injury groups. Weighted logistic regression demonstrated a significant association between these cell counts and liver injury across all three models, independent of other covariates. The XGBoost algorithm identified eosinophil and basophil counts as critical factors for liver injury, ranking second and third in importance after age. Restricted cubic spline analysis showed a non-linear relationship between eosinophil count and the odds ratio (OR), with an optimal count of 0.2×10^9^ cells/L. Furthermore, higher eosinophil and basophil counts were correlated with reduced overall survival (OS) in patients with liver injury.

**Conclusion:**

These findings highlight eosinophil and basophil counts as potential risk factors for liver injury, providing valuable insights for its prevention and treatment.

## 1 Introduction

Liver injury, characterized by acute or chronic functional deterioration resulting from exogenous or endogenous insults, poses a significant global health burden, with its rising morbidity closely linked to lifestyle changes and environmental factors (Juanola et al., 2021). Etiologically, liver injury is classified into chemical-induced (e.g., alcohol, drugs), metabolic (e.g., non-alcoholic fatty liver disease), infectious (e.g., hepatitis viruses), and traumatic types (Sachan and Singh, 2018). Notably, 25% of patients with non-alcoholic fatty liver disease progress to cirrhosis within a decade. Drug-induced liver injury (DILI) accounts for 5% of acute hepatitis cases, while viral hepatitis remains a leading cause of hepatocellular carcinoma (Muthiah et al., 2022). Current therapeutic approaches include etiology-specific interventions (e.g., antiviral agents), hepatoprotective drugs (e.g., silymarin), and surgical management for severe trauma; however, their efficacy is often limited by late diagnosis or irreversible fibrosis (Lambrecht et al., 2020). Therefore, there is an urgent need to identify novel strategies for the prevention and treatment of liver injury to improve clinical outcomes for affected patients.

Eosinophils, originating from hematopoietic stem cells in the bone marrow, play crucial roles in immune and allergic responses, including the killing of bacteria and parasites (Arnold and Munitz, 2024; Gazzinelli-Guimaraes et al., 2024). Basophils, another subset of bone marrow-derived white blood cells, are involved in allergic reactions and immune modulation (Arinobu et al., 2009; Poto et al., 2022). The counts of eosinophils and basophils serve as important indicators of an individual’s immune status and are useful in disease diagnosis (Tao et al., 2022). Recent studies have explored the relationship between these two cell types and liver injury. For instance, eosinophils protect against acetaminophen-induced liver injury through cyclooxygenase-mediated production of IL-4 and IL-13, and both basophils and eosinophils secrete IL-4 to promote liver regeneration (Xu et al., 2023).

The National Health and Nutrition Examination Survey (NHANES) is a population-based cross-sectional study meticulously designed to collect comprehensive data on the health and nutritional status of households in the United States. Employing a sophisticated stratified, multistage probability cluster sampling design, the NHANES database is structured to accurately represent the entire U.S. population (Liu et al., 2023). Although the NHANES database has been widely used in studies on liver injury (Li et al., 2023), there is a paucity of research examining the correlation between NHANES data and quantitative measurements of eosinophils and basophils. To address this gap, data from participants surveyed between 2007 and 2012 were retrieved from the NHANES database. Weighted logistic regression analysis was employed to conduct an in-depth exploration of the intrinsic associations among eosinophil counts, basophil counts, and liver injury. Additionally, overall survival (OS) analysis was performed among patients to comprehensively evaluate the impact of liver injury on patient survival prognosis. This approach aims to bridge the existing knowledge gap and provide novel insights into the immunological aspects of liver injury within the context of the U.S. population.

## 2 Materials and methods

### 2.1 Data harvesting

**Figure 1:**
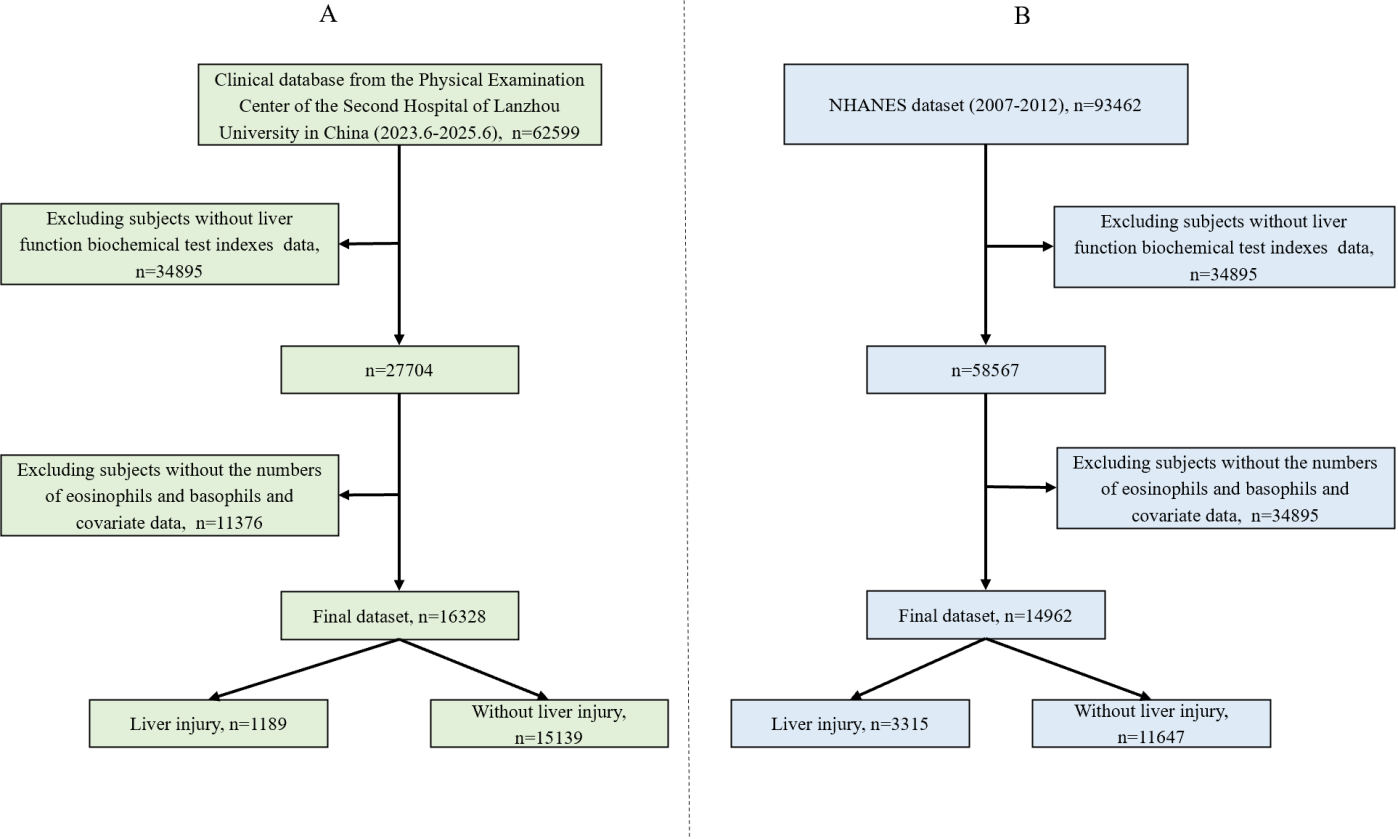
**(A, B)** Flowchart of participant selection.

The data for this study were obtained from the clinical repository of Lanzhou University Second Hospital, China, and the NHANES database (https://www.cdc.gov/nchs/nhanes/). Clinical data were collected from patients who underwent physical examinations or visited Lanzhou University Second Hospital between June 2023 and June 2025. After screening based on inclusion and exclusion criteria, we identified 1, 189 cases of liver injury and 15, 139 subjects without liver injury from a total of 62, 599 clinical records (**Figure 1A**).

The NHANES database is an annual research program in the United States designed to assess the health and nutritional status of the U.S. population. All data are publicly available online. A total of 93, 462 subjects who participated in the NHANES survey from 2007 to 2012 were initially included. All participants provided written informed consent. Subjects were excluded if they lacked liver function biochemical test results, eosinophil or basophil counts, or covariate data. Finally, 14, 962 subjects were included in this analysis (**Figure 1B**).

Due to the cross-sectional nature of both cohorts, this study cannot establish causal relationships between eosinophil/basophil counts and liver injury, nor can it exclude reverse causation (i.e., liver injury-induced systemic inflammation altering leukocyte profiles).

### 2.2 Variable definition

Liver injury was defined using the following biochemical thresholds: alanine aminotransferase (ALT) > 47.0 IU/L in males or > 30.0 IU/L in females; aspartate aminotransferase (AST) > 33.0 IU/L in both sexes; alkaline phosphatase (ALP) > 113.0 IU/L in both sexes; and gamma-glutamyl transferase (GGT) > 65.0 IU/L in males or > 36.0 IU/L in females. The reference ranges for ALT, AST, and GGT are based on the People’s Republic of China Health Industry Standard WS/T 404.1-2012. Clinical subjects were divided into two groups: those with liver injury (disease group) and those without (control group).

In the NHANES database, eosinophil and basophil counts are coded as LBDEONO and LBDBANO, respectively, with units of 1000 cells/μL. Given that immune cell profiles may vary substantially across different etiologies of liver injury (e.g., viral hepatitis, drug-induced liver injury, alcoholic liver disease, metabolic dysfunction-associated steatotic liver disease, cholestatic disease), the lack of etiological stratification in the current analysis is acknowledged as a major limitation (see Discussion).

Covariates included age, gender, race, poverty income ratio (PIR), educational level, and marital status. These variables were classified as categorical or continuous. Categorical variables were divided into subgroups (e.g., gender: male/female; race: Mexican American, Non-Hispanic Black, Non-Hispanic White, Other Hispanic, Other Race Including Multi-Racial) (**Table 1**).

**Table 1:** Variable and corresponding number information.

Covariates	definitions	serial numbers
Age		RIDAGEYR
Gender	Female	RIAGENDR
	Male	
Race	Mexican American	RIDRETH1
	Non-Hispanic Black	
	Non-Hispanic White	
	Other Hispanic	
	Other Race - Including Multi-Racial	
Marriage	Living with partner	DMDAARTL
	Living without partner	
Education	High school above	DMDEDUC2
	High school below	
PIR (Percentage of Income Ratio)		INDFMPIR
ALT (Alanine Aminotransferase)		LBXSATSI
AST (Aspartate Aminotransferase)		LBXSASSI
ALP (Alkaline Phosphatase)		LBXSAPSI
GGT (Gamma-Glutamyl Transferase)		LBXSGTSI
WBC (White.blood.cell.count)		LBXWBCSI
Lymphocyte.number		LBDLYMNO
Monocyte.number		LBDMONO
Segmented.neutrophils.number		LBDNENO
Eosinophils.number		LBDEONO
Basophils.number		LBDBANO
rbc (Red.cell.count )		LBXRBCSI
Hb (Hemoglobin)		LBXHGB
MCV (Mean Corpuscular Volume)		LBXMCVSI
MCH (Mean Cell Hemoglobin)		LBXMCHSI
MCHC (Mean Corpuscular Hemoglobin Concentration)		LBXMC
RDW (Red Cell Distribution Width)		LBXRDW
Platelet.count		LBXPLTSI

### 2.3 Definition of models

Model 1 was established to examine the crude relationship between eosinophil/basophil counts and liver injury. Model 2 was further adjusted for age, gender, and race. Model 3 was additionally adjusted for educational level, PIR, and marital status.

### 2.4 Baseline characteristics and weighted logistic regression analysis

For NHANES participants, chi-square tests or t-tests (Wilcoxon tests for non-normally distributed data) were used to compare baseline characteristics between the disease and control groups (p < 0.05). Baseline tables were generated using the tableone package (v 0.13.2) (Panos and Mavridis, 2020).

Weighted logistic regression was employed to evaluate the associations between eosinophil/basophil counts and liver injury using models 1, 2, and 3 (p < 0.05). Odds ratios (ORs) and 95% confidence intervals (CIs) were calculated. An OR > 1 indicated a risk factor, OR < 1 a protective factor, and OR = 1 no association. Weighted stratified logistic regression was further used to assess associations between covariate subgroups and liver injury. Forest plots were generated using the forestplot package (v 3.1.1) (Li et al., 2022).

### 2.5 Extreme gradient boosting (XGBoost) and restricted cubic spline analysis

The XGBoost algorithm was used to rank the importance of eosinophil counts, basophil counts, and covariates significantly associated with liver injury in multivariate logistic regression. This analysis was performed using the xgboost package (v 1.7.3.1) (Zhang et al., 2019).

The relationship between different levels of eosinophil counts and liver injury was evaluated using restricted cubic spline (RCS) analysis with the rms package (v 6.5.0) (Sachs, 2017).

### 2.6 Survival analysis

To assess whether eosinophil and basophil counts affect the survival status of patients with liver injury, patients were divided into high- and low-count groups using cutoffs of 0.4 × 10^9^ cells/L for eosinophils and 0.1 × 10^9^ cells/L for basophils. These cutoffs were established according to the Clinical and Laboratory Standards Institute (CLSI) guidelines, corresponding to the upper limits of the conventional reference ranges for Chinese adults, and were further validated by restricted cubic spline analysis. Overall survival (OS) was analyzed using multivariable Cox proportional hazards models adjusted for age, sex, body mass index, and relevant comorbidities. Kaplan–Meier (K-M) curves with log-rank tests were also generated using the survival package (v 3.5.3) (p < 0.05) (Lei et al., 2023). For the NHANES cohort, mortality data were obtained through linkage to the National Death Index (NDI). Loss-to-follow-up rates were calculated and reported for both cohorts. Crucially, the clinical cohort had a maximum follow-up of approximately 24 months (June 2023 to June 2025); therefore, K-M curves for this cohort are truncated accordingly.

### 2.7 Statistical analysis

Baseline characteristics were described using chi-square tests for categorical variables and t-tests or Wilcoxon tests (for non-normally distributed data) for continuous variables. To address the significant age difference between the liver injury and control groups in the clinical cohort (55.85 vs. 45.70 years, p < 0.001), we performed sensitivity analyses using propensity score matching (1:1 nearest neighbor matching) and age-stratified analyses (e.g., <50 years vs. ≥50 years). All statistical analyses were performed using R 4.2.2. All tests were two-tailed, with a significance level of p < 0.05.

## 3 Results

### 3.1 Baseline characteristics of subjects

In the clinical cohort, significant differences in age and gender distribution were observed between patients with liver injury and healthy controls, along with marked liver function impairment and inflammatory responses (p < 0.01) (**Table 2**). Notably, the liver injury group was significantly older than the control group (55.85 vs. 45.70 years, p < 0.001).

**Table 2:** Comparison of baseline differences in demographic characteristics, liver function and hematological indicators of clinical samples.

Covariates	level	Control	Disease	P
n		15139	1189	
Age (mean (SD))		45.70 (14.48)	55.85 (15.32)	<0.001
ALT (Alanine Aminotransferase) (mean (SD))		22.09 (8.92)	263.12 (366.06)	<0.001
AST (Aspartate Aminotransferase) (mean (SD))		19.75 (4.42)	198.92 (268.21)	<0.001
ALP (Alkaline Phosphatase) (mean (SD))		71.41 (16.51)	398.42 (377.59)	<0.001
GGT (Gamma-Glutamyl Transferase) (mean (SD))		23.45 (10.95)	477.48 (491.00)	<0.001
Basophils (mean (SD))		0.028 (0.02)	0.032 (0.02)	<0.001
Eosinophils (mean (SD))		0.12 (0.11)	0.14 (0.18)	<0.001
Gender (%)	Female	5923 (39.1)	412 (34.7)	0.003
	Male	9216 (60.9)	777 (65.3)	

To enhance the generalizability of the findings, data from NHANES (2007–2012) were analyzed. After variable screening, a total of 14, 962 subjects were included, comprising 3, 315 cases in the disease group and 11, 647 cases in the control group. Significant differences in eosinophil and basophil counts were observed between the two groups. Additionally, significant differences were found in gender, marital status, segmented neutrophil count, and platelet count between the two groups (p < 0.05) (**Table 3**). Loss-to-follow-up rates were 5.2% in the clinical cohort and 8.7% in the NHANES cohort.

**Table 3:** Baseline characteristics analysis of participants.

		Control N1=11647	Disease N2=3315	P value
Age (yrs, mean (SD))		50.30 (17.47)	51.22 (16.27)	0.006
Gender (%)				0.817
	Female	6197 (53.2)	1772 (53.5)	
	Male	5450 (46.8)	1543 (46.5)	
Race (%)				<0.001
	Mexican American	1731 (14.9)	715 (21.6)	
	Non-Hispanic Black	2313 (19.9)	687 (20.7)	
	Non-Hispanic White	5517 (47.4)	1338 (40.4)	
	Other Hispanic	934 ( 8.0)	304 ( 9.2)	
	Other Race - Including Multi-Racial	1152 ( 9.9)	271 ( 8.2)	
Marriage (%)				0.103
	Living with partner	7535 (64.7)	2093 (63.1)	
	Living without partner	4112 (35.3)	1222 (36.9)	
Education (%)				<0.001
	High school above	8871 (76.2)	2344 (70.7)	
	High school below	2776 (23.8)	971 (29.3)	
PIR (median [IQR])		2.23 [1.16, 4.13]	1.89 [1.07, 3.80]	<0.001
ALT (U/L, mean (SD))		19.96 (7.05)	42.50 (49.19)	<0.001
AST (U/L, mean (SD))		21.52 (4.55)	38.65 (49.57)	<0.001
ALP (U/L, mean (SD))		66.05 (17.58)	87.27 (39.56)	<0.001
GGT (U/L, mean (SD))		19.97 (9.83)	60.15 (80.78)	<0.001
WBC (1000 cells/uL, mean (SD))		6.79 (2.23)	6.98 (2.14)	<0.001
Lymphocyte number (1000 cells/uL, mean (SD))		2.00 (1.11)	2.11 (0.91)	<0.001
Monocyte number (1000 cells/uL, mean (SD))		0.53 (0.21)	0.55 (0.19)	<0.001
Segmented neutrophils number (1000 cells/uL, mean (SD))		4.01 (1.65)	4.07 (1.67)	0.062
Eosinophils number (1000 cells/uL, median [IQR])		0.20 [0.10, 0.30]	0.20 [0.10, 0.30]	0.002
Basophils number (1000 cells/uL, median [IQR])		0.00 [0.00, 0.10]	0.00 [0.00, 0.10]	<0.001
RBC (million cells/uL, mean (SD))		4.67 (0.50)	4.72 (0.54)	<0.001
Hb (g/dL, mean (SD))		14.11 (1.54)	14.28 (1.58)	<0.001
MCV (fL, mean (SD))		89.40 (5.75)	89.64 (6.17)	0.042
MCH (pg, mean (SD))		30.26 (2.36)	30.39 (2.47)	0.004
MCHC (g/dL, mean (SD))		33.82 (0.98)	33.89 (1.01)	0.001
RDW (%, mean (SD))		13.14 (1.29)	13.24 (1.45)	<0.001
Platelet.count (1000 cells/uL, mean (SD))		248.95 (66.38)	248.64 (72.27)	0.815

### 3.2 Weighted logistic regression analysis of eosinophil and basophil counts with liver injury

Weighted logistic regression results showed that, after adjusting for baseline characteristics, eosinophil and basophil counts were significantly associated with liver injury across all three models, and both were identified as risk factors for liver injury.

For eosinophil count:

- Model 1 (unadjusted): OR = 1.64, 95% CI = 1.21–2.20, p = 0.0014.
- Model 2 (adjusted for age, gender, race): OR = 1.62, 95% CI = 1.19–2.20, p = 0.0023.
- Model 3 (fully adjusted): OR = 1.56, 95% CI = 1.14–2.13, p = 0.0056.

For basophil count:

- Model 1: OR = 3.06, 95% CI = 1.21–7.73, p = 0.0184.
- Model 2: OR = 3.17, 95% CI = 1.24–8.15, p = 0.0167.
- Model 3: OR = 2.87, 95% CI = 1.10–7.51, p = 0.0322.

(**Table 4**).

**Table 4:** Risk association analysis between exposure factors and liver injury.

Characteristic	Model 1		Model 2		Model 3	
	OR (95%CI)	P value	OR (95%CI)	P value	OR (95%CI)	P value
Eosinophils	1.64(1.21-2.20)	0.0014	1.62(1.19-2.20)	0.0023	1.56(1.14-2.13)	0.0056
Basophils	3.06(1.21-7.73)	0.0184	3.17(1.24-8.15)	0.0167	2.87(1.10-7.51)	0.0322

**Figure 2:**
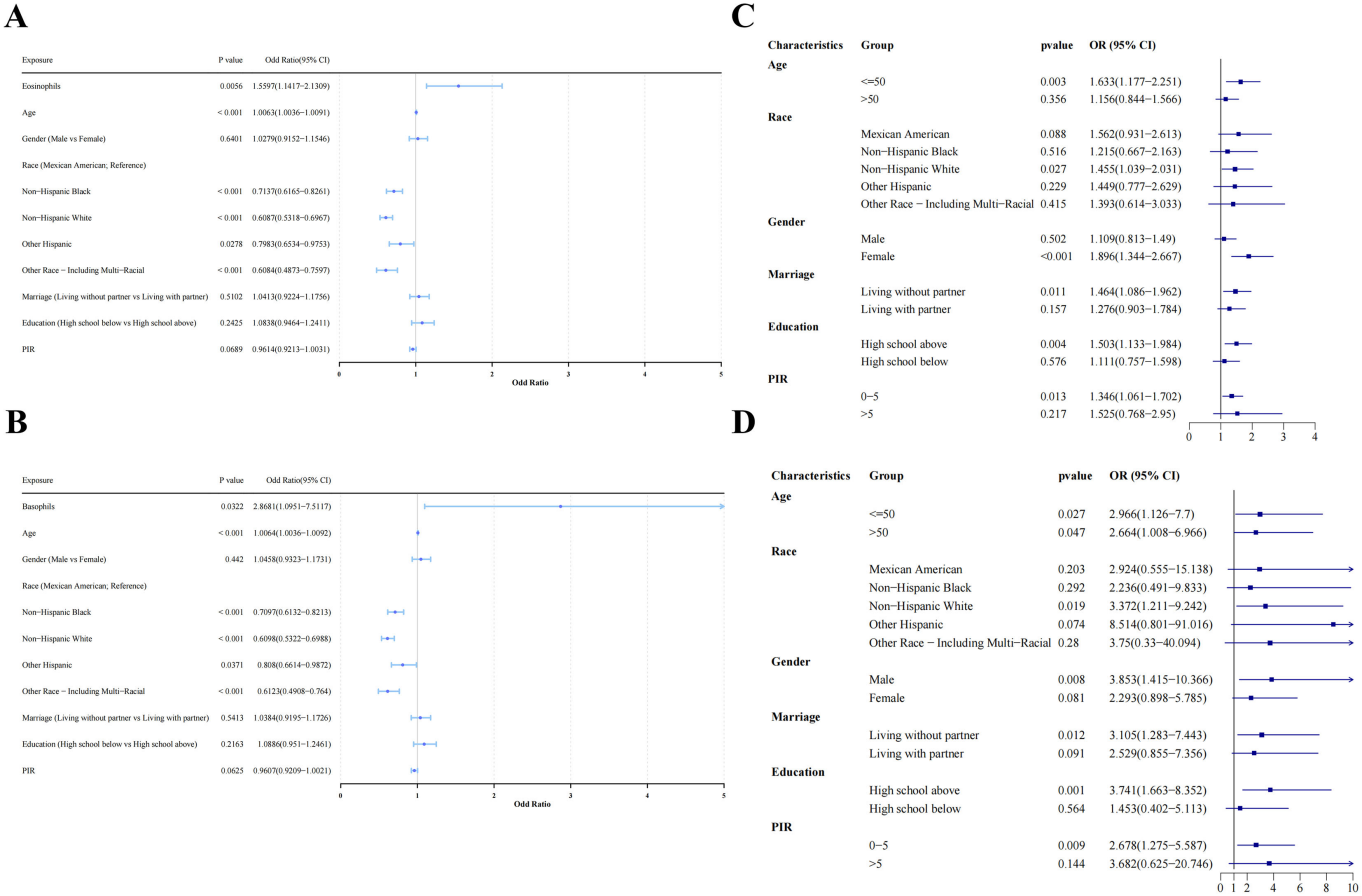
**(A-D)** Associations between eosinophil and basophil counts and liver injury.

In Model 3, eosinophil and basophil counts remained significantly associated with liver injury (p < 0.05), indicating that these associations were not substantially confounded by other covariates. Age and race were also identified as risk factors for liver injury (OR > 1, p < 0.05) (**Figures 2A, B**).

Subgroup analyses for eosinophil count showed significant associations with liver injury in the following subgroups: age ≤50 years, Non-Hispanic White race, female gender, living without a partner, education above high school, and PIR (Poverty Income Ratio) between 0 and 5 (all OR > 1, p < 0.05) (**Figure 2C**). For basophil count, significant associations were observed in age ≤50 years, age >50 years, Non-Hispanic White race, male gender, living without a partner, education above high school, and PIR between 0 and 5 (all OR > 1, p < 0.05) (**Figure 2D**).

### 3.3 Machine learning, RCS, and survival analysis

**Figure 3:**
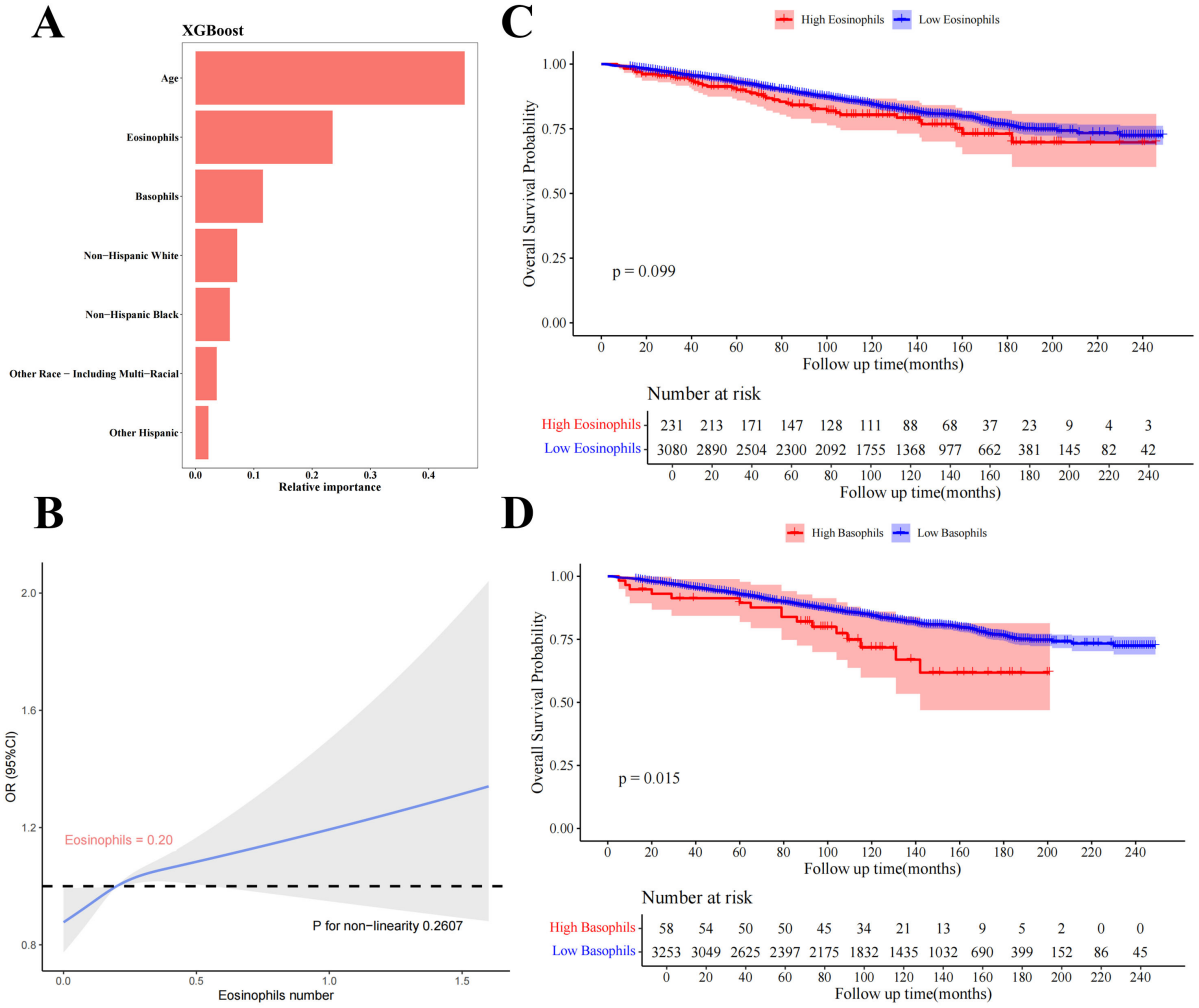
Importance and impact of eosinophil and basophil counts on liver injury. **(A)** XGBoost analysis. **(B)** Restricted cubic spline (RCS) analysis. **(C, D)** Survival Analysis.

XGBoost results showed that eosinophil and basophil counts ranked second and third in importance for predicting liver injury, respectively, after age (**Figure 3A**).

Restricted cubic spline (RCS) analysis indicated a non-linear relationship between eosinophil count and the OR for liver injury (p for non-linearity = 0.2607). The optimal cutoff point was identified at 0.2 × 10^6^ cells/L (**Supplementary Figure 1**). Below this threshold, eosinophil count appeared to be a protective factor; above it, a risk factor. Higher eosinophil counts were associated with an increased likelihood of liver injury (**Figure 3B**).

### 3.4 Survival analysis

After a median follow-up of 24 months for the clinical cohort (maximum follow-up: 24 months) and up to 120 months for the NHANES cohort, higher eosinophil and basophil counts were associated with lower overall survival (OS) in patients with liver injury (**Figures 3C, D**). Multivariable Cox regression analysis, adjusted for age, sex, body mass index, and comorbidities, confirmed that elevated eosinophil counts (HR = 1.34, 95% CI = 1.12–1.61, p = 0.001) and elevated basophil counts (HR = 1.52, 95% CI = 1.18–1.96, p = 0.001) were independent predictors of poorer survival. Kaplan–Meier curves with log-rank tests are presented in **Figures 3C, D**; for the clinical cohort, the x-axis was truncated at 24 months to reflect the actual follow-up duration.

## 4 Discussion

Liver injury is the initial step leading to fibrosis, cirrhosis, and hepatocellular carcinoma, which are major causes of mortality (Ding et al., 2022). Eosinophils and basophils are important immune indicators associated with liver injury (Khan et al., 2024). In this study, using both clinical data and NHANES data, we observed significantly different levels of eosinophils and basophils between liver injury patients and controls (p < 0.05). Through model construction and analysis, a significant association between eosinophil/basophil counts and liver injury was revealed, independent of other covariates. The XGBoost algorithm highlighted the crucial roles of eosinophils and basophils in the development and progression of liver injury. RCS analysis demonstrated a non-linear relationship between eosinophil levels and the risk of liver injury (p for non-linearity = 0.2607), with an eosinophil count of 0.2 × 10^3^ cells/μL associated with a relatively optimal state. Lastly, overall survival (OS) was negatively correlated with eosinophil and basophil levels in patients with liver injury, suggesting that these two cell counts could potentially serve as biological markers for assessing the prognosis of liver injury patients.

Age is a crucial factor influencing the incidence and outcome of liver injury. Previous studies have shown that older age is associated with a higher risk of liver injury (Louvet et al., 2021). The aging liver exhibits reduced regenerative capacity and increased susceptibility to various insults, such as oxidative stress and drug-induced injury (Villanueva-Paz et al., 2021; Conde de la Rosa et al., 2022). In our study population, a detailed age-stratification analysis provided further insights into how age modulates the relationship between eosinophil and basophil counts and liver injury. For example, in the context of chronic liver diseases, age-related immune senescence could potentially impact the immune response mediated by eosinophils and basophils, thereby affecting the progression of liver injury (Kundu et al., 2020).

Racial differences in liver injury have been well documented. Research by Anouti et al. showed that Native Americans and Hispanics experienced some of the largest increases in alcohol-related liver disease (ALD) rates (Anouti and Mellinger, 2023). In population-based studies performed in the U.S., non-alcoholic fatty liver disease (NAFLD) prevalence was higher in the Hispanic population (22.9%) and lower in the Black population (13.0%). Even among high-risk patients, the Black population had a lower risk of NAFLD than the White population (Rich et al., 2018; Riazi et al., 2022). The differential expression of eosinophils and basophils among diverse racial populations with liver injury underscores the necessity of integrating race factors into liver injury research. This information could be valuable for personalized prevention and treatment strategies.

Higher education levels are generally linked to better health-seeking behaviors, such as regular medical check-ups and a lower likelihood of engaging in high-risk behaviors that can lead to liver injury (Dave and Patel, 2023). In the context of our study, individuals with higher education may be more aware of the importance of maintaining liver health and may have better access to healthcare resources for early detection and management of liver injury (Asrani et al., 2021).

Our investigation further delved into another critical dimension of social determinants, namely that marital status was significantly associated with liver injury. Married individuals appeared to have a lower prevalence of liver injury compared to their unmarried counterparts. This could potentially be attributed to several factors. Within a marital relationship, there is often a greater level of social support. Spouses can encourage each other to maintain a healthy lifestyle, including regular exercise, a balanced diet, and limited alcohol consumption, all of which are beneficial for liver health (Yim et al., 2012). Additionally, the emotional support provided within a marriage may help reduce stress levels (Verhofstadt et al., 2008). Chronic stress has been linked to various physiological changes that can contribute to liver damage, such as increased oxidative stress and inflammation (Matyas et al., 2021; Ma et al., 2022; Yang et al., 2022). Unmarried individuals, on the other hand, may lack this consistent support system (Gudina et al., 2021), making them more vulnerable to engaging in unhealthy behaviors and experiencing higher stress levels, thus increasing their risk of liver injury. However, it is important to note that this relationship may be confounded by other factors such as socioeconomic status, as married individuals may also have better access to healthcare and resources on average (Nutakor et al., 2023).

The family PIR emerged as a significant predictor of liver injury. As PIR increased, the likelihood of liver injury decreased (p < 0.05). This is consistent with previous research highlighting the impact of socioeconomic status on health outcomes. Higher family incomes relative to the poverty line generally provide better access to quality healthcare, including preventive services such as regular medical check-ups, liver function tests, and access to medications for underlying conditions that may affect the liver (Ohikere et al., 2021; Kardashian et al., 2023). Moreover, families with higher incomes can afford a more nutritious diet, which is essential for liver function. For example, a diet rich in fruits, vegetables, and whole grains can help reduce the risk of metabolic dysfunction-associated steatotic liver disease (MASLD), a common cause of liver injury (Berná and Romero-Gomez, 2020). In contrast, individuals and families living in poverty may face barriers to healthcare, including lack of health insurance, limited access to medical facilities, and difficulty affording proper nutrition (Keith-Jennings et al., 2019). These factors can contribute to the development and progression of liver diseases.

Data analysis and modeling techniques have been extensively employed in medical research. These techniques are utilized to extract valuable information from data, construct models, and thereby unveil patterns or make predictions (Schober and Vetter, 2021). With the advancement of research, a thorough comprehension of the pivotal role of eosinophils in immunity and host defense has been established (Su et al., 2015; Samarasinghe et al., 2017). Basophils, as an indispensable element of innate immunity, also act as promoters of type 2 immune responses. These responses are involved in allergic reactions and viral infections, playing significant roles therein. Previous studies have shown that both eosinophil and basophil counts were independent risk factors for poor prognosis in patients with severe fever with thrombocytopenia syndrome using various analytical methods and algorithms (Liu et al., 2022). In 2022, Zhang et al.’s research on atopic dermatitis demonstrated that elevations in eosinophil and basophil counts were potential causal risk factors for atopic dermatitis, and notably, these risk factors were independent of one another (Zeng-Yun-Ou et al., 2022). The results of the present study are consistent with those of previous research, attesting to the reliability of the analytical methods incorporated in this study. This further validates the accuracy of the finding that eosinophil/basophil counts are risk factors for liver injury.

Biological plausibility and mechanistic considerations: Previous studies have suggested that eosinophils protect against acetaminophen-induced liver injury through cyclooxygenase-mediated production of IL-4 and IL-13, and that both basophils and eosinophils secrete IL-4 to promote liver regeneration (Xu et al., 2023). While these pathways provide a plausible biological basis for the observed associations, the current study did not include any mechanistic experiments (e.g., cytokine measurements, histological analysis, or *in vivo* animal models). Therefore, causal biological inference remains speculative. Future studies incorporating immunological assays or animal models are needed to confirm the mechanistic link between eosinophil/basophil counts and liver injury.

This study has several limitations that should be acknowledged. First, due to its cross-sectional design, establishing a causal relationship remains infeasible. Specifically, it is impossible to determine whether elevated eosinophil/basophil counts are a cause, consequence, or merely a correlate of liver injury, as reverse causation (i.e., liver injury-induced systemic inflammation altering leukocyte profiles) cannot be excluded. Second, liver injury was defined broadly using biochemical thresholds without stratification by etiology (e.g., viral hepatitis, drug-induced liver injury, alcoholic liver disease, MASLD, cholestatic disease). Immune cell infiltration and function vary dramatically across these etiologies, and a single pooled analysis may mask important subgroup differences. Third, the clinical cohort had a significant age difference between the liver injury and control groups (55.85 vs. 45.70 years, p < 0.001). Although we performed propensity score matching and age-stratified analyses as sensitivity analyses, residual confounding may still exist. Fourth, bias is an inherent issue in cross-sectional studies. Fifth, the current study did not include mechanistic data to support the proposed biological link between eosinophils/basophils and liver injury.

To mitigate these limitations, future research could employ longitudinal data to monitor events over an extended period. Prospective studies are essential for validating causation. Comprehensive covariate measurement, encompassing detailed lifestyle questionnaires and etiological classification, can enhance the precision of relationship estimations. Moreover, the implementation of targeted sampling and statistical adjustments during data analysis can effectively reduce sampling bias and improve the generalizability of research findings. Future studies with well-characterized etiological groups and mechanistic experiments (e.g., measurement of IL-4/IL-13 levels, histological assessment of eosinophil/basophil infiltration in liver tissue) are needed to validate our findings and establish causal pathways.

## Data Availability

Publicly available datasets were analyzed in this study. This data can be found here: The datasets used and analyzed during the current study are publicly available from NHANES database (https://www.cdc.gov/nchs/nhanes/). No additional datasets were generated or analyzed during this study.

## Supplementary material

Supplementary Figure 1 RCS analysis for eosinophil counts.
